# Neoadjuvant treatment of melanoma: case reports and review

**DOI:** 10.1186/2162-3619-2-30

**Published:** 2013-11-08

**Authors:** Shachar Laks, Kevin A Brueske, Eddy C Hsueh

**Affiliations:** 1 Columbia Surgical Associates, Columbia, Missouri and the Division of General Surgery, Saint Louis University, St. Louis, Missouri, USA; 2 Department of Surgery, Saint Louis University, 3635 Vista at Grand Blvd., St. Louis, Missouri 63110, USA

**Keywords:** Metastatic melanoma, Vemurafenib, Ipilimumab, Neoadjuvant therapy

## Abstract

Neoadjuvant therapy is an under-utilized regimen for the treatment of metastatic melanoma. The use of this approach has been increasing in other tumor types. Neoadjuvant therapy may reduce occult circulating tumor cell burden in the face of bulky disease and afford a real time evaluation of treatment effectiveness. Neoadjuvant approach can also provide preoperative histologic and molecular analysis of treated tissue that may guide the postoperative treatment planning in patients with resectable metastatic melanoma lesions. The putative benefits of better margin control and clearance of occult systemic disease would theoretically improve surgical outcome. With the advent of effective agents against metastatic melanoma, this common approach to the treatment of rectal cancer, metastatic colon cancer, and breast cancer should also be evaluated as a viable treatment strategy for advanced stage melanoma.

## Introduction

Malignant melanoma is a highly curable cancer when detected early but often fatal at advanced stage. In 2010, the prevalence of melanoma in the United States was 921,780 affected individuals. It was estimated that 76,690 additional people would be diagnosed with melanoma and 9,480 will die of their melanoma in 2013. It has a predilection to affect younger Americans in the prime of their lives. In fact, it is the most common form of cancer in young adults ages 25 to 29 years old, and the second most common cancer in those ages 15 to 24 years old. [1] Melanoma has a further predilection for higher socioeconomic groups and has an estimated annual productivity loss due to mortality of $3.5 billion [2]. Most of the melanoma cases diagnosed are in early stages of disease and have an excellent prognosis with 86-95% 10 year survival with appropriate therapy. Unfortunately, more advanced disease carries a poor prognosis with American Joint Commission on Cancer (AJCC) stage IIIB/C patients having a 24-43% 10 year survival, and stage IV disease with a 10-15% 5 year survival [3].

Currently, the mainstay of therapy for melanoma has been surgical resection to render the patient clinically free of disease for both early and select advanced stage melanoma patients. For patients with AJCC stage I and II melanoma, surgical therapy would entail wide excision with or without sentinel lymph node dissection depending on the Breslow thickness of the primary lesion and presence of other significant prognostic variables. For patients with AJCC stage III melanoma patients with nodal involvement, the therapy would involve radical lymphadenectomy. For patients with resectable AJCC stage IV disease, metastasectomy for limited burden disease would be the usual treatment of choice. Until recently chemotherapeutic, biologic, and immunologic therapies have had little success in the adjuvant setting with only high dose interferon (HDI) having an FDA indication in the adjuvant setting.

Over the last 4 years, the advent of targeted therapy for BRAF mutated melanoma and immune checkpoint inhibitors, e.g. anti-CTLA-4, anti-PD-1, and anti-PD-1 L antibodies have sparked resurgence of excitement for the treatment of advanced stage melanoma. With significant portions of advanced stage melanoma patients harboring occult systemic disease as demonstrated by subsequent relapse after surgery and presence of CTC in peripheral blood, neoadjuvant therapy could potentially improve surgical outcome for metastatic melanoma patients with resectable lesions. Furthermore, response to neoadjuvant therapy has been demonstrated to be an important prognostic variable in several tumor types, such as breast and colorectal cancer. The objective response to neoadjuvant therapy would likely serve as an important prognostic variable for stratification of care following surgery for metastatic melanoma patients. More importantly, this approach would allow selection of patients that would most likely benefit from surgery. Patients who progressed on systemic therapy would likely have biologically aggressive histology where early relapse can be expected after radical resection of metastatic disease. As promising agents are being developed and approved for treatment in metastatic melanoma over the last several years, [4] there have been scant reports of neoadjuvant treatment using these new agents. To allow an objective comparison of the upcoming neoadjuvant regimens with these new agents, we reviewed the literature for neoadjuvant treatment of melanoma with the traditional regimens [Table 1] and case reports of this approach with the recently approved agents. To highlight the promise and potential issues of neoadjuvant therapy for metastatic melanoma, we also report herein our experience with post-treatment resection of residual metastatic disease for patients with previously unresectable stage IV melanoma receiving systemic therapy as primary treatment modality.

**Table 1:** Summary of traditional neoadjuvant studies

	**Patients (n)**	**Study design, end point**	**Agent**	**Clinical response**	**Survival**
Sasson et al. [5]	16, metastatic	Retrospective, OS	Various*	62.5% (7PR, 3CR)	OS – 68.8% DFS – 62.5%, median f/u 35 mo
Jouary et al. [6]	13, metastatic	Retrospective, OS	DTIC	60% did not progress.	OS - 31.6 vs 25.3 mo (study group vs. retrospective cohort)
Shah et al. [7]	19, Stage III	Phase II, ORR	Tem	16% (1PR, 2CR)	NR
Buzaid et al. [8]	64, Stage III	Phase II, ORR	Cis, Vin, IL2, DTIC, IFN	50%**(28PR, 4 CR)	Median OS 27 mo. Median DFS 13 mo
Gibbs et al. [9]	48, Stage III	Phase II, ORR	Cis, Vin, IL2, DTIC, IFN	38.9%^#^ (13PR, 1CR)	79% OS, 65% PFS at 2.6 yrs.
Koyanagi et al. [10]	63, Stage III	Phase II, DFS	Cis, Vin, DTIC, IL2, IFN	NR	DFS- 70%, median f/u 30.4 mo. 2 yr OS – 80.9%
Lewis et al. [11]	92, Stage III	Phase II, OS	Cis, Vin, DTIC, IL2, IFN	26%^##^	RFS – 64% OS- 78%, median f/u 40.4 mo
Kounalakis et al. [12]	153, Stage III	Retrospective, OS	Cis, Vin, DTIC, IL2, IFN	55% ^**+**^ (14 PR, 14 CR)	5 yr OS-82% (micromet disease), and 77% (bulky disease)
Moschos et al. [13]	20, Stage III	Phase II, ORR	HDI	55% (8PR, 3 CR)	90% PFS at 1.5 yrs.

*Study designed to compare utility of resection vs. no resection and had various chemotherapeutic regiments including: single agent DTIC or combination with camustine, cis-platin, tamoxifen, or interferon.

**Reported histological response rate.

^#^Only 36 of the 48 patients had clinically evaluable disease to assess response rates.

^##^Only 50 of the 92 patients had clinically evaluable disease to assess response rates.

^**+**^Only 51 of the 153 patients had clinically evaluable disease to assess response rates.

## Case reports

**Figure 1:**
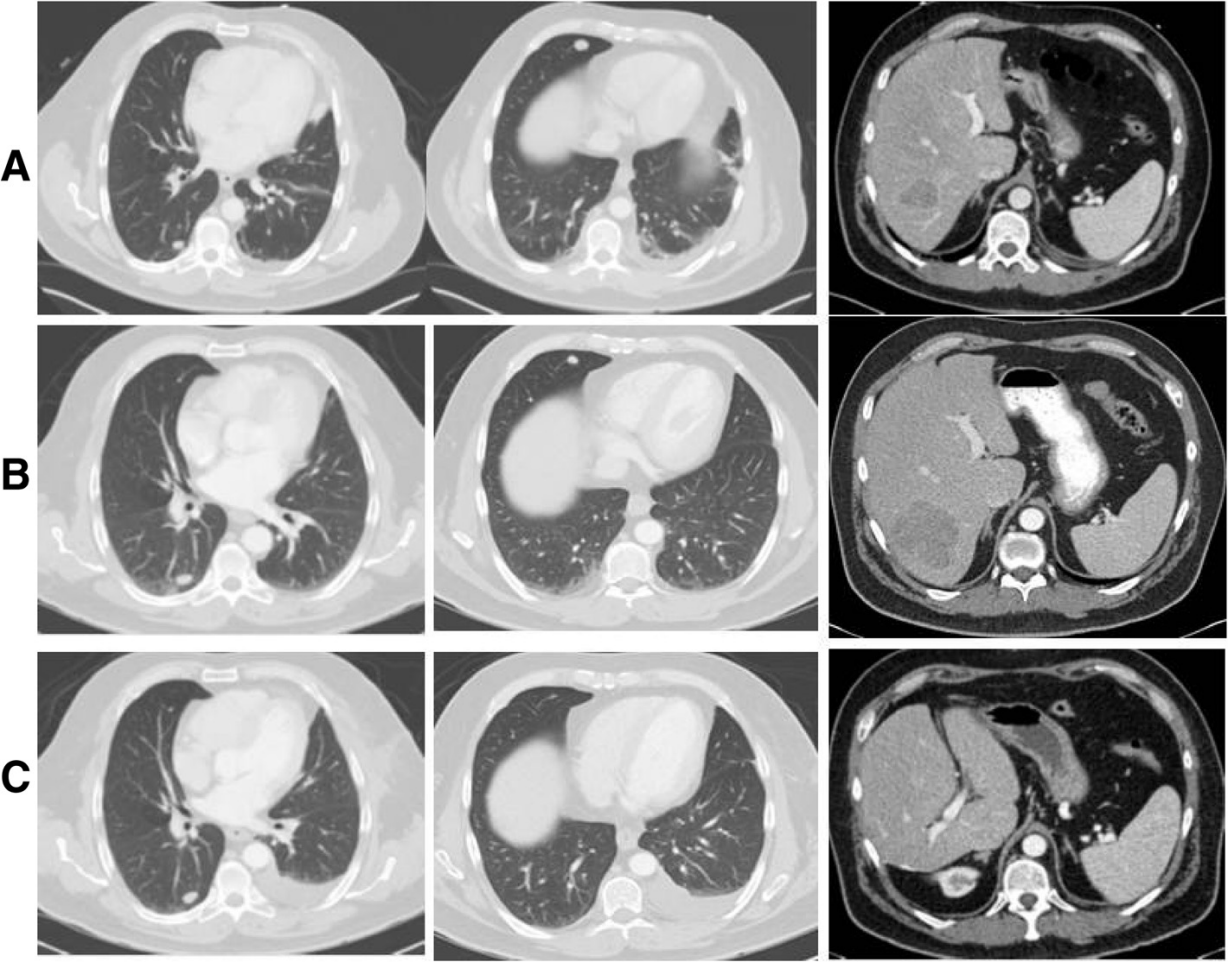
**Pre- and post-treatment CT images for patient 1. A**. Initial metastatic disease. Small pulmonary nodules on the right anterior and posterior lungs and a 5.4 cm lesion in the right lobe of the liver. **B**. Post-treatment. Stability or reduction in sizes of right lung lesions, but progression of right lobe of liver lesion to 7 cm. **C**. Post right hepatectomy. Continued regression and resolution of right lung lesions, and surgical resolution of right liver lesion.

Our first case is a 55 years old male with a 1.3 mm thick non-ulcerated nodular melanoma on the forehead. Patient underwent wide excision and sentinel node biopsy for treatment of his primary melanoma. At year 4 of post-operative surveillance, patient recurred with pulmonary and hepatic metastases [Figure 1A]. He underwent high-dose IL2 followed by ipilimumab (3 mg/kg for 4 doses every 3 weeks) for progression of disease. At 6 months post-ipilimumab treatment, mixed responses were observed in the pulmonary lesions with stable disease in some and regression in others. However, the right lobe of liver lesion increased in size from 5.4 to 7 cm [Figure 1B]. A right hepatectomy for a 7 cm metastatic melanoma with negative margins was performed. Postoperative surveillance revealed no further disease in the liver and continued regression or stabilization of the lung nodules [Figure 1C]. Follow up PET scanning 4 months later revealed new left chest wall and axillary recurrence and patient underwent en bloc resection of the axillary and chest wall diseases to negative margins. He remains progression free on surveillance scans 4 months after resection of his chest wall disease.

**Figure 2:**
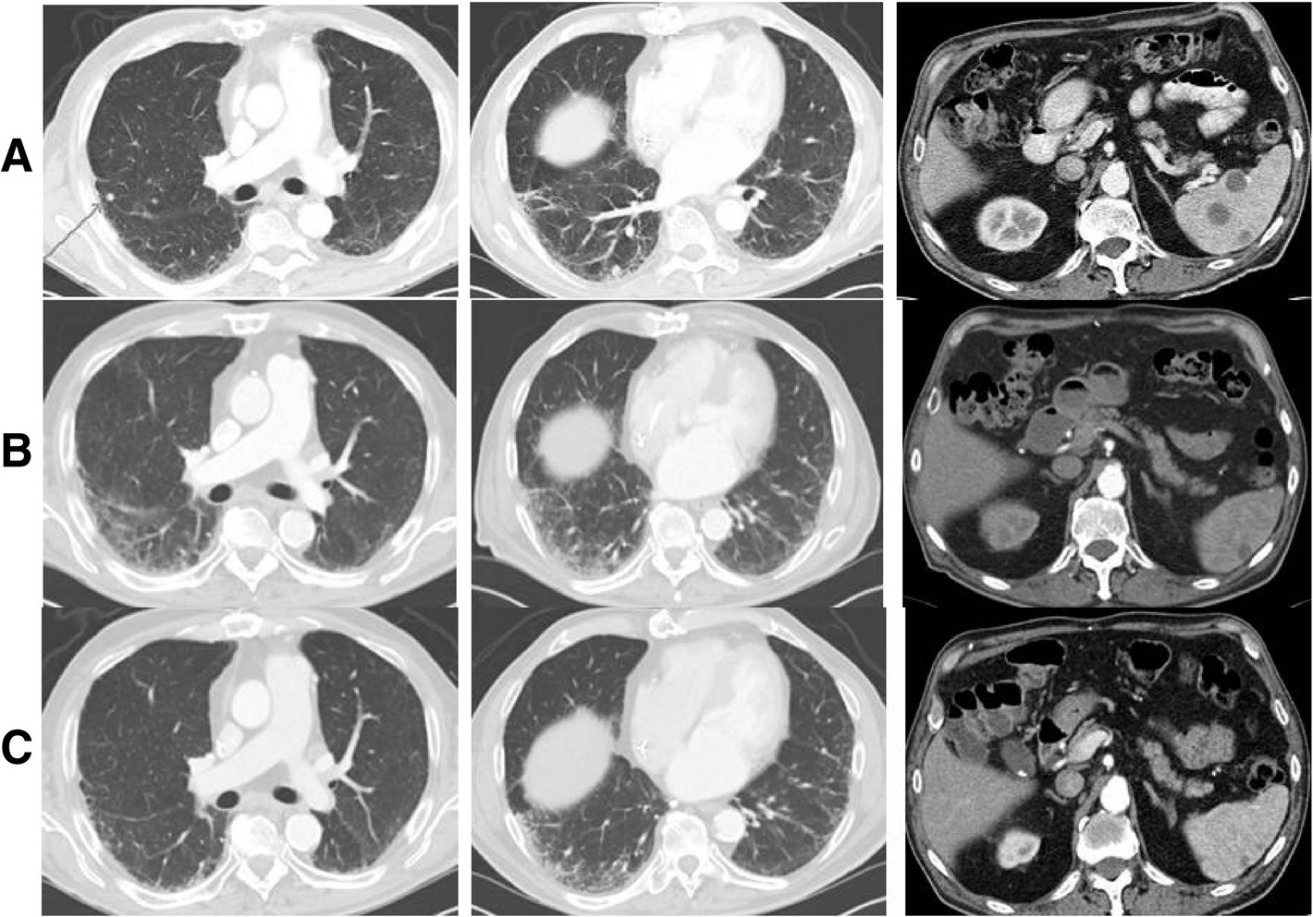
**Pre- and post-treatment CT images for patient 2. A**. Pre-treatment CT images of metastatic lung nodules and splenic metastases. **B**. Post-treatment CT images of resolving lung and splenic metastases. **C**. Twenty month post-treatment CT images of resolved lung and splenic metastases.

**Figure 3:**
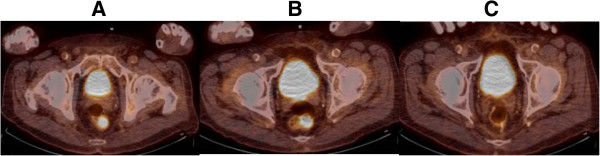
**Pre- and post-treatment PET/CT images for patient 2. A**. Pre-treatment PET/CT image of rectal lesion. **B**. Post-treatment PET/CT image of rectal lesion. **C**. Post-resection PET/CT image of rectal lesion.

Our second patient is an 81 year old male with a 2.9 mm thick non-ulcerated right ear primary melanoma. Patient underwent a wide excision and sentinel lymph node with negative surgical margin and negative sentinel lymph node. Eight months post-op, patient recurred in the draining nodal basin and underwent a right neck dissection with 6 of 24 positive lymph nodes and right parotidectomy with 4 of 4 positive intra-parotid lymph nodes. Patient subsequently recurred distantly 2 months later with PET/CT revealing metastatic lung disease, splenic metastases, and a focus of disease in the rectum [Figures 2A and 3A]. Standard ipilimumab therapy was initiated. Post treatment surveillance revealed resolution of lung and splenic disease, but persistence of rectal uptake [Figures 2B and 3B]. Patient underwent low anterior resection of the rectal lesion with viable tumor on histologic analysis. He is in complete remission at one year follow up [Figures 2C and 3C].

### Neoadjuvant therapy with traditional systemic agents

For decades, single-agent dacarbazine (DTIC) has been the standard systemic therapy for metastatic melanoma and the control arm for most of the promising therapeutic intervention trials. As a single agent, objective response rate is approximately 15% with median response duration of 5 to 6 months and rare complete responders [14]. Given the poor performance of single agent DTIC, various phase I and Phase II trials of combination regimens such as BOLD (bleomycin, vincristine, lomustine, and DTIC), CVD (cisplatin, vinblastine, and DTIC), and “Dartmouth regimen” (cisplatin, carmustine, DTIC, and tamoxifen) have been tested and some have shown response rates as high as 55%. However, no statistical significant impact on overall survival can be demonstrated with multiple randomized phase III trials of various combination therapy versus DTIC alone [14]. Boddie et al. reported their experience of neoadjuvant DTIC followed by resection in four children with stage II-IIIB melanoma [15]. All four patients were alive at follow up of between 2–10 years. This compared favorably with the historical 35% survival rate in childhood melanoma at the time. Sasson et al. reported their single institution experience with neoadjuvant therapy in 16 metastatic melanoma patients [5]. Various regimens including single agent DTIC or combination with camustine, cis-platin, tamoxifen, or interferon were administered. All 16 patients underwent resection. They observed a median survival of 35 months compared with 11.5 months in a contemporaneous non-resection control. Since the comparison variable was surgical resection versus no resection, no inference can be made about the utility of neoadjuvant approach. Jouary et al. reported their single institution retrospective review of single agent DTIC in the neoadjuvant setting for melanoma pulmonary metastases [6]. In their cohort of 13 patients, they observed a median survival of 31.6 months versus 25.3 months in the no neoadjuvant group. They noted a survival benefit for those patients who had stable or regressed disease during neoadjuvant treatment compared with those who had progressive disease. Shah et al. reported their phase II neoadjuvant temozolomide trial in resectable stage III and IV patients [7,16]. They observed a 16% response rate, not significantly different from the 12-14% response rate seen in the widely metastatic group. Of the 16%, 2 had a complete response, 1 had a partial response, and 4 had stable disease. Janco et al. reviewed their institutional data of 50 vaginal/vulvar melanoma resections with a poor median overall survival of 3.3 years and a 30.9% 5 year survival [17]. One patient received temozolomide for vaginal cancer, but died 3 months after surgery from progressive disease, while one vaginal and one vulvar melanoma received carboplatin and paclitaxel with bevacizumab and were both disease free and alive at 2 and 5 years follow up.

Another regimen tested in the neoadjuvant setting for melanoma was biochemotherapy. Biochemical therapy typically consisted of vinblastine, cisplatin, DTIC, IL-2, and interferon alpha. In a neoadjuvant study with 64 stage III melanoma patients, Buzaid et al. reported a histologic response rate of 50% [8]. Four of the treated patients achieved complete histologic response. Median overall survival was 27 months and median disease-free survival was 13 months. Gibbs et al. reported their neoadjuvant biochemotherapy phase II study with 36 stage III patients [9]. An objective response of 38.9% was observed with 1 complete response and 13 partial responses. Of interest, 14 of the remaining patients had minor response. Median overall survival and progression-free survival were not reached. At median follow-up of 31 months, 79.2% of patients were alive and 65% were progression-free. Koyangi et al. utilized circulating tumor cells to monitor the effects of neoadjuvant biochemotherapy in 63 patients [10]. At median follow-up of 30.4 months, 70% of the patient cohort was clinically disease free. Lewis et al. reported a multicenter phase II neoadjuvant biochemotherapy trial in stage III melanoma patients [11]. At 42 months of follow up, a relapse free survival of 64% and overall survival 78% were achieved. However, only 26% clinical response rate was observed. To ensure the safety of this aggressive approach, Kounalakis et al. reviewed their single institution experience with 153 patients and observed no significant increase in wound complication rates or lymphedema with this approach [12]. They also reported a 5 year overall survival rate of 77% in patients with clinical adenopathy. Despite early excitement, a phase III trial of 138 patients comparing biochemotherapy vs. high dose interferon in the adjuvant setting was stopped early because of futility at the interim analysis with median follow up of 49.3 months [18].

In 1986, Creagan et al. investigated the use of interferon alfa in disseminated malignant melanoma [19]. Interferon alfa was shown to have a 22% overall response rate with 3 complete responses. Median progression-free survival was 1.5 months and overall survival was 5 months. Moschos et al. reported the results of a phase II neoadjuvant HDI therapy in 20 patients with stage IIIB disease [13]. A 55% clinical response rate was observed. They also noted the correlation of response to neoadjuvant therapy and clinical outcome with mean disease-free survival of 32 months for the responders and 10 months for the non-responders. This observation corroborated the findings in numerous neoadjuvant trials in other tumor types. Despite early excitement, no phase III trial of neoadjuvant HDI has shown improved survival.

### Neoadjuvant therapy with recently approved agents

#### Vemurafenib

Mutations in BRAF lead to constitutive activation of downstream signaling of the RAS-RAF-MEK-ERK (mitogen-activated protein kinase) signal transduction pathway in 40-60% of cutaneous melanoma with substitution of glutamic acid for valine at codon 600 in 90% of the BRAF-mutated melanoma [20,21]. In vivo, vemurafenib suppresses ERK signaling and hence tumor cell proliferation and survival in mutant BRAF melanoma, but lacks activity in wild-type BRAF melanoma cell lines [22-25]. In a phase III randomized trial of vemurafenib versus DTIC in 675 patients with BRAF V600E mutated previously untreated metastatic melanoma patients, overall survival was 84% for vemurafenib group and 64% for DTIC group at 6-month analysis [26]. Despite significant initial clinical response to mutant BRAF inhibition, resistance soon develops and disease progresses. Resistance to vemurafenib seems to be related to reactivation of the MAP Kinase pathways [27]. Trametinib is an inhibitor of this pathway, and has been shown to have an effect on BRAF mutated melanoma [28]. In an open label phase I/II trial, combination BRAF and MEK inhibition with dabrafenib and trametinib (respectively) improved progression free survival from 5.8 months with dabrafenib monotherapy to 9.4 months with combination therapy in metastatic melanoma patients with BRAF V600 mutation [29]. Response rate with combination therapy was 76% in comparison to 54% with monotherapy (p = 0.03). An increased duration of response of 10.5 months vs. 5.6 months was also observed.

With the impressive responses observed in vemurafenib trials, several investigators have attempted to use BRAF inhibition in the neoadjuvant setting. Fadaki et al. reported a case of a 58 year old with bulky stage IIIC unresectable melanoma from the left axilla and neck that they treated with 4 months of vemurafenib therapy with an impressive clinical and radiographic response to allow resection of the disease [30]. Following neck and axillary dissections, only 1 microscopic foci of viable tumor in 40 lymph nodes was noted. Tumor necrosis changes were observed in the other nodes. The patient also received adjuvant vemurafenib, and was disease free at 6 month follow up at the publication date. Koers et al. reported a 47 year old man who presented with bulky axillary and supraclavicular disease matted to the surrounding skin and chest wall from unknown primary site [31]. Patient was treated with vemurafenib with resulting radiological and clinical response followed by radical resection. Histological analysis of surgical specimen revealed only minimal residual viable tumor with negative margins. Patient was disease free at time of the report. Kolar et al. reported the use of neoadjuvant vemurafenib in a patient with a large symptomatic brain metastasis that was initially not amenable to resection [32]. Following clinical response with vemurafenib treatment, resection of brain metastasis was performed with negative margins and no viable tumor left in the specimen. Patient underwent postoperative radiation and had no evidence of local recurrence at 12 month follow up.

#### Ipilimumab

Another novel agent recently approved for the treatment of metastatic melanoma is ipilimumab, a fully human monoclonal antibody that blocks cytotoxic T-lymphocyte-associated antigen 4 (CTLA-4), an immune checkpoint molecule. CTLA-4 plays a key role in the suppression of T cell activation [33-35]. CTLA-4 blockade has been shown to decrease the regulatory affect and increase downstream mediators of the immune response such as IL2, IL10, and interferon [36,37]. Weber et al. reported their phase II study using ipilimumab in 115 treatment-naïve melanoma patients with unresectable stage III or IV disease [38]. Patient were randomized to ipilimumab at 10 mg/kg every 3 weeks for 4 doses with or without budesonide. They observed a best overall response rate (BORR) of 12% in budesonide group and 15.8% in placebo group. The disease control rate (DCR) was 31.0% and 35.1%, respectively.

Hodi et al. reported the initial phase III study with 676 patient with unresectable stage III or stage IV disease who had progressed on other therapies [39]. Patients were randomized in a 3:1:1 fashion to ipilimumab with or without gp100 vaccine and vaccine alone. The 3 mg/kg dosage of ipilimumab was used. They observed an improvement of overall survival with 6.4 months in the vaccine alone group and 10 months in the ipilimumab groups. An overall response rate to ipilimumab of 29.5% was observed with 60% of responses lasting over 2 years. Further inhibition of other checkpoint molecules appeared to increase clinical response. Wolchok et al. reported a phase II study with combination of ipilimumab and nivolumab (an antibody against the programmed death 1 [PD-1] receptor) given in a concurrent or sequential fashion [40]. Patients in the concurrent regimen had a 40% clinical response rate compared with 20% in the sequential regimen group. At the maximum doses with acceptable side effects, 53% clinical responses were observed. Hamid et al. reported the results of another anti-PD-1 antibody, lambrolizumab, in 135 previously treated metastatic melanoma patients including those patients who progressed on ipilimumab [41]. Objective response rate of 38% was observed. Responses were durable with median follow up of 11 months for patients who had responses.

Although no neoadjuvant experience with Ipilimumab has been reported, Gyorki et al. reported their immunologic observation in 23 patients undergoing surgery within 30 days or receiving the treatment during the induction or maintenance phase of Ipilimumab therapy [42]. Only grade 1 or 2 wound complications were observed in 22% of the patient cohort. There were no grade 3–5 complications. In 10 patients with available matched tumor specimen and peripheral blood samples, significantly higher percentage of CD4 + FOXP3+ T-regulatory cells and lower ratio of CD8+/CD4 + FOXP3+ in the tumor compared with blood were reported. Safety of preoperative ipilimumab therapy has also been evaluated in 12 surgical patients with urothelial carcinoma of the bladder [43]. Only grade I/II toxicities were observed. The University of Pittsburgh is enrolling patients in a neoadjuvant study of ipilimumab in the setting of resectable stage IIIB/C patients, and have released some initial data in regards to immune monitoring and clinical response, but the final analysis of this trial are eagerly awaited [44].

### Other reported uses of neoadjuvant therapy in melanoma

Radiotherapy has been shown to be effective in the adjuvant setting in advanced stage III melanoma by Intergroup Randomized Trial (TROG 02.01/ANZMTG 01.02) [45]. Foote et al. reported their experience with neoadjuvant radiation in 12 patients with histologically proven stage III melanoma [46]. Following radiotherapy, restaging work up revealed progression of disease in 2 patients and resection was not attempted. Ten patients underwent lymphadenectomy with local disease control rate of 92% at 1 year and 1 year disease free survival of 54%. Surgical complications were minor. Based on these encouraging results, the investigators are conducting a multicenter phase II study.

Mozzillo et al. reported the neoadjuvant use of electrochemotherapy in a patient with isolated large subcutaneous metastasis to the cheek [47]. Based on clinical evaluation, patient would have required a complex and disfiguring resection for clearance of disease. Following a regimen of intravenous bleomycin and local tumor ablation with electroporation (Cliniporator) for 2 cycles they were able to obtain significant tumor shrinkage. Patient underwent an excision with simple local reconstruction three months after the second treatment with no viable tumor on histologic analysis of the surgical specimen. Patient remained disease free at 1 year follow up.

## Discussion

Melanoma is an increasingly prevalent disease that has a predilection for the young and productive members of our society with an immeasurable social, economic, and emotional cost. The disappointing historical results of melanoma systemic therapy are rapidly replaced with promising effective therapeutics targeting multiple pathways in tumor growth and immune suppression. While the initial studies of novel agents were tested as single agent in the metastatic settings as proof of principle, the most effective way to treat metastatic melanoma may encompass targeting tumor growth, reversing tumor immune suppression, and cytoreduction of clinical tumor bulk. Neoadjuvant approach is historically under-utilized in the treatment of metastatic melanoma in comparison to other tumor types mainly due to the ineffectiveness of systemic therapy. The promising results of biochemotherapy neoadjuvant trials and the emergence of active agents in melanoma suggested that this approach would shed light on the rational selection of optimal treatment for metastatic melanoma patients.

We have presented two cases of post-ipilimumab resection of residual disease in patients with metastatic melanoma. Others have reported their case reports with post-vemurafenib resection of residual disease. These case series were not considered “neoadjuvant” in the typical sense, i.e. surgery was only contemplated after treatment rendered the patients surgical candidates. The case reports cited herein demonstrates the potential and most apparent benefit of neoadjuvant therapy for metastatic melanoma where tumor bulk shrinkage was achieved with systemic therapy followed by margin-negative resection. It is yet to be demonstrated, as with other tumor types, that neoadjuvant therapy would improve survival as compared with upfront radical surgery followed by adjuvant therapy. The two cases reported herein illustrated the other potential benefit and challenge facing the design of neoadjuvant therapy for melanoma. Since melanoma readily metastasizes to multiple organ sites, neoadjunvant treatment may allow the selection of optimal surgical patient cohort where no new lesions appear during treatment. As demonstrated in our first patient, although growth of liver lesion was observed during treatment, there was regression and stabilization of pulmonary lesions with no new lesions developed during treatment. With the increasing evidence showing the efficacy of immune checkpoint therapy for melanoma and the prolonged response time reported, the determination of optimal timing of surgery would be instrumental in the design of neoadjuvant therapy with this class of agents.

With the recent breakthrough in melanoma treatment and availability of multiple effective agents, the choice of agent or agents and the timing of treatment sequence would be crucial for successful outcome in designing a neoadjuvant treatment for metastatic melanoma. While targeted therapy such as BRAF and MEK inhibitors can effect early shrinkage of tumor bulk increasing the resectability of target lesions, the durability of response with this class of treatment has not been uniformly reported and may not have significant impact on overall survival. Conversely, immune checkpoint therapy can confer a durable disease control but tumor shrinkage is protracted several months and responses in multiple lesions may not be uniform, i.e. progression in some lesions and regression in others. Identification of optimal patient population and optimal combination for neoadjuvant approach and the concurrent biomarker studies would further advance our treatment of metastatic melanoma patients. With limited data available in the neoadjuvant treatment of melanoma with these new agents, we are eagerly awaiting the results of the University of Pittsburgh trial of ipilimumab in the stage IV setting.

### Consent

Written informed consent was obtained from the patients for the publication of this report and any accompanying images.

## Competing interests

The authors declare that they have no competing interests.

## Authors' contributions

All authors contributed equally to this manuscript. All authors read and approved the final manuscript.
